# Fibromodulin Interacts with Collagen Cross-linking Sites and Activates Lysyl Oxidase*

**DOI:** 10.1074/jbc.M115.693408

**Published:** 2016-02-18

**Authors:** Sebastian Kalamajski, Dominique Bihan, Arkadiusz Bonna, Kristofer Rubin, Richard W. Farndale

**Affiliations:** From the ‡ Department of Laboratory Medical Sciences, Lund University, Medicon Village 406-3, 22363 Lund, Sweden and; the § Department of Biochemistry, Downing Site, University of Cambridge, Cambridge CB2 1QW, United Kingdom

**Keywords:** collagen, extracellular matrix, fibromodulin, lysyl oxidase, small leucine-rich proteoglycan (SLRP), cross-linking

## Abstract

The hallmark of fibrotic disorders is a highly cross-linked and dense collagen matrix, a property driven by the oxidative action of lysyl oxidase. Other fibrosis-associated proteins also contribute to the final collagen matrix properties, one of which is fibromodulin. Its interactions with collagen affect collagen cross-linking, packing, and fibril diameter. We investigated the possibility that a specific relationship exists between fibromodulin and lysyl oxidase, potentially imparting a specific collagen matrix phenotype. We mapped the fibromodulin-collagen interaction sites using the collagen II and III Toolkit peptide libraries. Fibromodulin interacted with the peptides containing the known collagen cross-linking sites and the MMP-1 cleavage site in collagens I and II. Interestingly, the interaction sites are closely aligned within the quarter-staggered collagen fibril, suggesting a multivalent interaction between fibromodulin and several collagen helices. Furthermore, we detected an interaction between fibromodulin and lysyl oxidase (a major collagen cross-linking enzyme) and mapped the interaction site to 12 N-terminal amino acids on fibromodulin. This interaction also increases the activity of lysyl oxidase. Together, the data suggest a fibromodulin-modulated collagen cross-linking mechanism where fibromodulin binds to a specific part of the collagen domain and also forms a complex with lysyl oxidase, targeting the enzyme toward specific cross-linking sites.

## Introduction

Dense collagen matrix in tendons and fibrotic collagen matrix in tumors and atherosclerotic plaques is enriched in a collagen-associated protein, fibromodulin (FMOD)^5^ (1–7). This protein is a member of SLRPs whose temporospatial expression patterns vary in different tissues and whose absence, in the respective knockout mice, creates tissue-specific collagen matrix phenotypes. Their differential expression and interactions with collagen are attributed to the different ways collagen matrices can be organized (reviewed in Refs. 8, 9). FMOD itself influences collagen fibrillogenesis and cross-linking. *Fmod*^−/−^ mouse tendons have a disturbed collagen fibril phenotype (1, 10), and *Fmod*^−/−^ tendon collagen has a lower acid extractability, excessively oxidized α1(I)-C-telopeptides, and aberrantly cross-linked α2(I)-chains (11). Also, the morphological phenotype of *Fmod*^−/−^ tendon collagen fibrils resembles the phenotype of fibrils where lysyl oxidase (LOX), the major collagen cross-linking enzyme, is inhibited (12). FMOD might therefore be involved in directing dense collagen cross-linking in tendons and fibrotic matrices, although the exact mechanisms are unknown.

Collagen cross-linking depends on LOX and at least one of its homologues, LOXL2, which catalyze the oxidation of specific collagen lysines (13–18). Through oxidation, lysines acquire reactive aldehydes that condense with other lysines, resulting in an initial cross-link formation that becomes more complex as the collagens assemble into fibrils (19). LOX is activated through BMP-1 processing of its inactive pro-isoform, a process potentiated by fibronectin (20) and further amplified by BMP-1 itself being incorporated into the fibronectin matrix through interaction with periostin (21). When activated, LOX oxidizes lysine residues in collagen telopeptides (22, 23) while binding to the collagen triple-helical domain (24). This process is more efficient on fibrillar rather than monomeric collagen (25, 26), suggesting that proteins that specifically modulate collagen fibrillogenesis could also influence the specificity of LOX-mediated cross-linking. Through excessive cross-linking that imparts stiffness on the collagen matrix, LOX enhances fibrosis-enhanced metastasis (27–29). Despite the dependence of collagen cross-linking on the dynamics of fibril assembly and its restriction to specific collagen domains, research on potential modulators of these processes, especially in relation to LOX, has been scarce. Interestingly, LOX is not a collagen-exclusive enzyme but can oxidize other substrates, *e.g.* elastin, PDGF receptor, and TGF-β (30–32), raising the question of whether specific mechanisms exist that target LOX to collagen. Hypothetically, such proteins could modulate LOX activity in the extracellular space during the assembly of collagen fibrils.

In this paper, we hypothesized that FMOD could influence LOX activity near the collagen cross-linking sites through binding to the specific collagen domains and/or through modulating LOX activity. To investigate this hypothesis, we mapped the collagen-binding sites of FMOD and tested and mapped the potential FMOD-LOX interaction. We also analyzed LOX quantity and distribution in *Fmod*^−/−^ tendons and assayed LOX activity under the influence of FMOD. Our data, together with previously published findings, strongly suggest that FMOD can directly modulate site-specific LOX-mediated collagen cross-linking.

## Experimental Procedures

### 

#### 

##### Materials

Collagen Toolkit peptides and their substituted variants were synthesized on TentaGel R Ram resin as C-terminal amides using either an Applied Biosystems Pioneer or CEM Liberty peptide synthesizer, as described previously (33, 34).

The collagen Toolkits II and III contain 56 or 57 peptides, each with 27 residues of primary collagen sequence with neighboring peptides overlapping by nine amino acids, thus spanning the whole of the corresponding collagen domain. The peptides are flanked by GPP_(5)_ repeats and GPC triplets unless stated otherwise. These features impart a stable triple-helical conformation on the entire peptide.

Collagen was extracted from mouse tail tendons using 20 mm acetic acid. Recombinant lysyl oxidase protein used in interaction assays was purchased from Origene. For transient expression of lysyl oxidase in 293T cells for use in LOX activity assays, LOX cDNA was ligated into the p3xFLAG-CMV-8 vector (Sigma), replacing the native signal peptide sequence with the secretion pre-pro-trypsin leader sequence. Antibodies against lysyl oxidase (EPR4025) and against the His tag (18184) were from Abcam. Antibodies against fibromodulin were from Sigma (SAB1100690).

##### Reagents

Biotin EZ-link reagent (ThermoFisher) was used to biotinylate recombinant fibromodulins using the instructions of the manufacturer, quenched with ethanolamine, and dialyzed against TBS. Streptavidin-HRP was from Millipore. The 3,3′,5,5′-tetramethylbenzidine substrate kit was from Pierce.

##### Protein Expression and Purification

His tag-modified recombinant fibromodulin (human, amino acids 19–376) and its fragments (FMOD90, amino acids 31–376; FMODNT, amino acids 19–95) were expressed in 293T cells using the pCEP4 vector (Invitrogen). Proteins were purified on Ni-NTA-Sepharose (Qiagen) and further on Superose 6 (GE Healthcare) in PBS. Bacterial His-tagged FMOD was expressed in BL21 *Escherichia coli* using the pET27(b) vector (Novagen) and purified as follows. Cells were lysed in 8 m urea in 100 mm NaH_2_PO_4_ and 100 mm Tris (pH 8.0) (lysis buffer). The lysate was cleared by sonication and centrifugation, and the supernatant was incubated with Ni-NTA-Sepharose. The Sepharose was then washed in lysis buffer (pH 6.3), and fibromodulin was eluted in lysis buffer (pH 8.0) containing 250 mm imidazole. Fibromodulin was then dialyzed against PBS with 10% glycerol and gradually decreasing urea concentrations (from 8 to 0 m). In the final step, the protein was purified on size exclusion chromatography using PBS. All steps were performed at 4 °C. All protein identities were confirmed by mass spectrometry.

##### Collagen-binding Assays

Each assay included full-length collagen as a positive control and BSA and a GPP_(10)_ triple-helical peptide, like the Toolkit flanking sequence, as negative controls. Collagen peptides were coated at 10 μg/ml in 20 mm acetic acid overnight at 4 °C. Plates were rinsed three times with TBS and blocked with 5% BSA in TBS for 1 h at room temperature. After blocking, plates were incubated with biotinylated fibromodulin at 10 μg/ml in TBST with 0.1% BSA for 1 h. After washing with TBST, streptavidin-HRP was added at 1:10,000 dilution in TBST 0.1% BSA and incubated for 1 h. After washing with TBST, the binding was detected with TMB substrate and stopped with 2 m sulfuric acid, and absorbance was read at 450 nm.

Assays where fibromodulin binding to coated collagen I was tested in the presence of Toolkit peptides were performed using similar methods, but here acetic acid-extracted tail tendon mouse collagen I was diluted to 10 μg/ml into PBS, distributed into 96-well plate wells, incubated at 37 °C for 1 h to induce fibril formation, and then incubated at 4 °C overnight for coating. Fibromodulin (10 μg/ml) was preincubated for 2 h with Toolkit peptides of different concentrations before incubating with the coated collagen.

##### Solid-phase Binding Assays

Either lysyl oxidase or fibromodulin or its variants were coated overnight on a 96-well plate at 5 μg/ml in sodium carbonate buffer (pH 9.2). Collagen was coated at 10 μg/ml in PBS. The remaining part of the assay followed the protocol described above (collagen-binding assays), but here binding of lysyl oxidase to coated fibromodulin proteins or collagen was detected with rabbit anti-lysyl oxidase and HRP-conjugated anti-rabbit antibody, and binding of fibromodulin proteins to coated lysyl oxidase or collagen was detected with mouse anti-His tag and HRP-conjugated anti-mouse antibodies.

##### Immunohistochemistry

Sections of paraffin-embedded tail tendons from wild-type and *Fmod*^−/−^ mice were used. Antigen retrieval solution (L.A.B. solution from Polysciences) was used before quenching peroxidase with 0.3% hydrogen peroxide for 15 min and blocking the sections with 10% goat serum in TBS for 1 h. Slides were incubated with anti-fibromodulin (2 μg/ml in TBS and 1% serum) for 2 h, washed with TBS, and stained with the ultrasensitive ABC rabbit IgG staining kit (Thermo Scientific) and diaminobenzidine peroxidase substrate kit (Vector Labs).

##### Immunoblots

Guanidine-extracted proteins from wild-type and *Fmod*^−/−^ tendons were dissolved in urea/CHAPS solution and quantified using a BCA assay (Thermo Scientific). 30 μg of total protein was run on 4–12% BisTris SDS-PAGE reducing gels (Thermo Scientific). Proteins were transferred to a nitrocellulose membrane that was immunoblotted using 5% milk in TBS for blocking, TBS with 0.1% Tween 20 for washing, and washing buffer with 0.5% milk for antibody incubations. Anti-lysyl oxidase was used at 1 μg/ml, and HRP-conjugated anti-rabbit was used at 0.2 μg/ml. The antibodies were detected with SuperSignal West Dura (Thermo Scientific) and imaged using a charge-coupled device camera.

##### Lysyl Oxidase Activity Assays

Fresh conditioned medium (293 serum-free expression medium, Life Technologies) from 293T cells transiently expressing recombinant lysyl oxidase (from the p3xFLAG-CMV-8 vector) or a mock control was used. Cells were transfected with either vector using Lipofectamine 3000, and medium was collected after 3 days. Aliquots of medium were incubated with recombinant fibromodulin or its fragments for 1 h before mixing with lysyl oxidase reaction buffer (lysyl oxidase activity assay kit, Abcam). Samples were incubated for 15 min at 37 °C, and LOX activity was measured by fluorescence detection 530/590 nm excitation/emission.

##### Proximity Ligation Assays

Duolink proximity ligation assays were purchased from Sigma and performed according to the instructions of the manufacturer. Here we used HFL-1 cells seeded on coverslips and transfected with FMOD-pCEP His-tagged constructs (described above) or, as a negative control, His-tagged non-collagen binding domain of FMOD (leucine-rich repeat domains 1–3). His-tagged proteins were detected with a mouse anti-His tag. Endogenous LOX was detected with rabbit anti-LOX. Secondary antibodies coupled to DNA probes were included in the proximity ligation assay kit.

## Results

### 

#### 

##### Interaction of Fibromodulin with Collagen Toolkit Peptides

**FIGURE 1.**
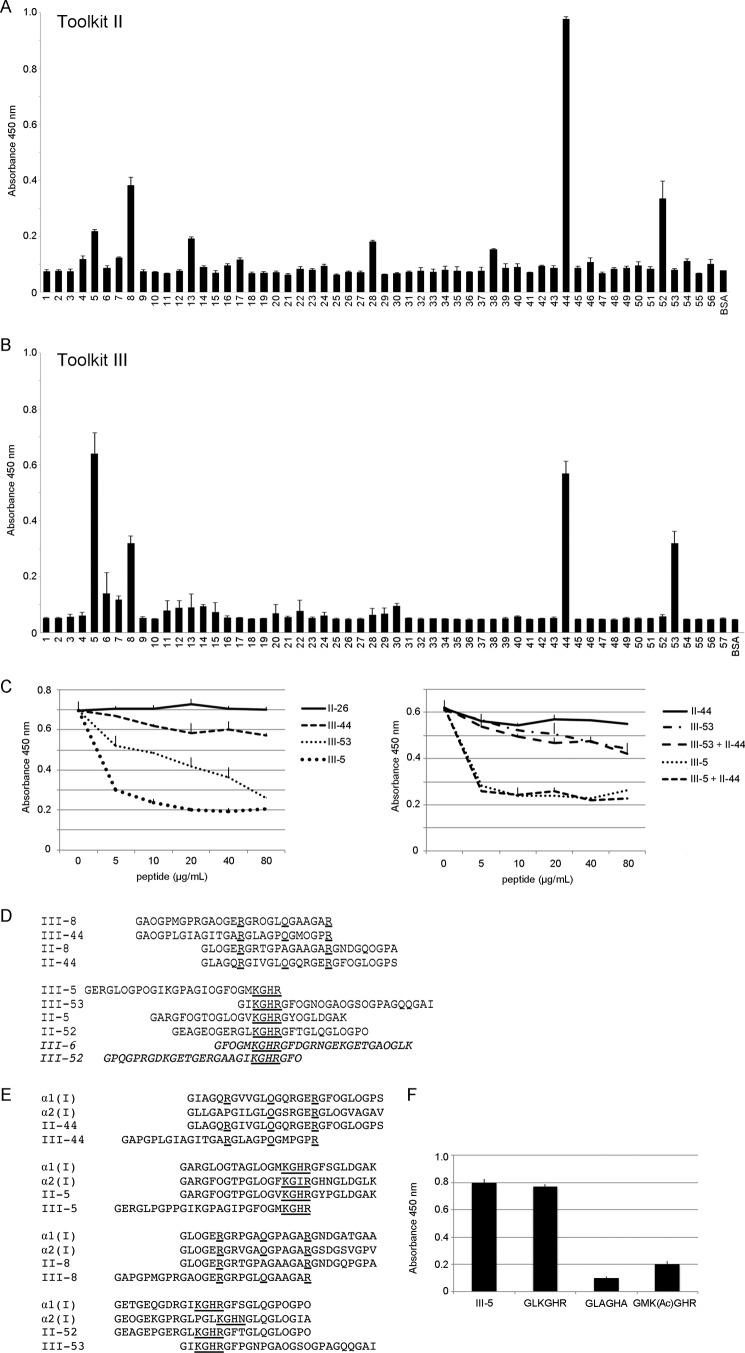
**Identification of fibromodulin binding sites on collagen.** *A* and *B*, collagen Toolkit II (*A*) and III (*B*) peptides were coated on a 96-well plate. The plate was blocked with BSA and incubated with biotinylated FMOD at 10 μg/ml (170 nm) for 2 h. Binding was detected with streptavidin-HRP followed by TMB substrate, quenching with sulfuric acid, and measuring *A*_450_. BSA was used as a negative control. *C*, inhibition of FMOD binding to coated collagen I. The assay was performed as above, but collagen I was coated on a 96-well plate, and FMOD was preincubated with increasing amounts of the interacting Toolkit peptides III-5, III-44, and III-53 or with a negative control peptide, II-26. *D*, sequences of the interacting Toolkit peptides are listed, with homologue amino acids *underlined* (except the repetitive glycines). The non-binding peptides are shown in *italics. E*, homology alignment of FMOD-binding Toolkit peptides and the corresponding sequences on collagen I α1 and α2 chains. *F*, solid-phase binding assay to assess interaction of FMOD with cross-linking site-containing collagen peptides and their mutated variants. The assay was performed as in *A*. Peptide sequences are listed on the *x* axis (*K(Ac)*, acetylated lysine), except the III-5 sequence, which is listed in *E.* These shorter peptides were synthesized with GCP terminal triplets. All *error bars* represent mean ± S.D. (*n* = 3 technical replicates). All experiments were performed three times.

Because *Fmod*^−/−^ tendons have altered collagen cross-linking (11), we hypothesized that FMOD could modulate collagen cross-linking by interacting with specific cross-linking sites. To identify the binding sites, we used collagen II and III Toolkits in solid-phase assays. The assays showed FMOD affinity for the helix cross-linking sites (peptides II-5, III-5, II-52, and III-53 in both Toolkits; Fig. 1 *A* and *B*; both Toolkits were used contemporaneously). Other peptides, II-44 and III-44, containing the collagenase cleavage site or the peptide II-8 earlier identified as an MMP interaction site (35, 36) also had a high apparent affinity for FMOD (Fig. 1, *A* and *B*). Finally, FMOD had no affinity for telopeptides (data not shown).

To validate our data, we tested the efficiency of Toolkit peptides in inhibiting FMOD-collagen type I interaction. We selected the peptides with the highest apparent affinity for FMOD, *i.e.* II/III-44, III-5, and III-53, along with a negative control peptide, II-26. In a solid-phase binding assay, the cross-linking site, peptides III-5 and III-53, could efficiently inhibit this interaction, whereas II/III-44 were only weak inhibitors, even at a 30-fold molar excess over FMOD (Fig. 1*C*). Combining II-44 with III-5 or III-53 did not result in a higher inhibitory effect than when using these peptides alone (Fig. 1*C*, *right panel*). Also, III-53 showed an inconsistent inhibitory effect in different experiments, whereas III-5 and II/III-44 inhibitions gave similar results.

Sequences of the interacting Toolkit peptides are listed and aligned in Fig. 1*D*. Among the peptides that contain the known helix cross-linking sites, the KGHR motif was the most obvious conserved sequence, making it a likely fibromodulin-binding site. Regarding peptides III-8, II-44, and III-44, a homologous pattern of arginine and hydroxyproline residues could be identified as a second, lower-affinity, putative fibromodulin-binding site (Fig. 1*D*). This charge distribution is the only one occurring in Toolkits II and III. Aligning the II/III-44 peptides with the respective sites on collagen I α chains shows a high degree of conservation in the α1(I) chain (only two conservative substitutions) and α2(I) chain (slightly less conserved) (Fig. 1*E*). Regarding the helical cross-linking sites, they are all conserved in collagens I, II, and III.

To test our hypothesis that FMOD interacts with the helical collagen cross-linking site, we tested the binding of FMOD to a peptide containing only this site (GLKGHR) and compared the binding with two mutated variants. As Fig. 1*F* shows, the interaction with the cross-linking site is high and depends on lysine. FMOD does not bind when lysine is alanine-substituted or acetylated. Also, arginine or histidine alone cannot compensate for the absence of lysine.

##### Fibromodulin Interactions with Lysyl Oxidase

**FIGURE 2.**
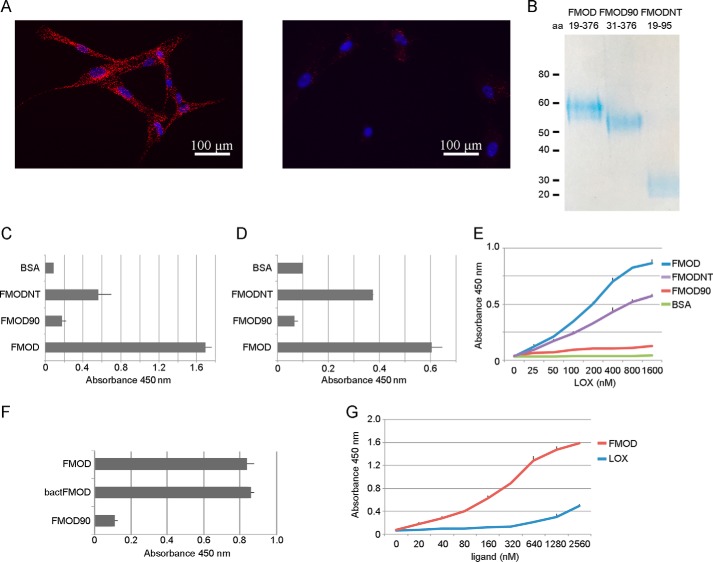
**Identification and characterization of the FMOD-LOX interaction.** *A*, proximity ligation assay on HFL-1 cells expressing FMOD (*left panel*) or non-collagen-binding His-tagged FMOD fragment as a negative control (*right panel*). Anti-His tag and anti-LOX antibodies were used to detect the interaction (*red fluorescence*). Nuclear staining with DAPI is shown in *blue. B*, gel image of FMOD variants used in the solid-phase assays in *C–F*. The proteins were expressed in 293T cells and purified on Ni-NTA-Sepharose. The amino acid (*aa*) range of each construct is listed. *C*, solid-phase assays showing interactions between FMOD variants and LOX. LOX was coated on a 96-well plate that was blocked with BSA and incubated with 170 nm FMOD variants, whose binding was detected using anti-His tag antibody. *D*, as in *C*, but here FMOD variants were coated on the plate followed by incubation with 150 nm LOX and detection with anti-LOX antibody. The experiments in *C* and *D* were repeated twice, giving similar results. *E*, as in *D*, but here serial dilutions of LOX were used to determine the apparent *K_D_*. Similar results were obtained in three experiments. *F*, solid-phase assay to assess binding of LOX to coated FMOD (expressed in 293 cells), bactFMOD (expressed in *E. coli*), and FMOD90. The assay was performed as in *D. G*, solid-phase assay to compare binding of serially diluted FMOD and LOX to coated collagen in a 96-well plate. Binding was detected using anti-His tag or anti-LOX antibodies. All *error bars* represent mean ± S.D. (*n* = 3 technical replicates). The experiments in *F* and *G* gave similar results on three occasions.

Because of the collagen cross-linking phenotype in *Fmod*^−/−^ mice, we also hypothesized that FMOD may interact with LOX, an interaction that could modulate the accessibility of LOX to collagen and its cross-linking sites. To identify potential FMOD-LOX interactions, we performed proximity ligation assays on HFL-1 fibroblasts expressing His-tagged FMOD or a His-tagged non-collagen-binding fragment of FMOD (negative control) and endogenous LOX. The assays detected an interaction between FMOD and LOX (Fig. 2*A*).

We wanted to identify the binding site of fibromodulin to LOX. Because we earlier identified the collagen-binding site to be in the LRR domain (37), we hypothesized that LOX binding could be in the N-terminal domain of FMOD. We therefore made a recombinant N-terminal fragment of FMOD (FMODNT, amino acids 19–95), and truncated FMOD (FMOD90, amino acids 31–376) where we deleted the 12 N-terminal amino acids. We expressed the proteins in 293T cells, purified them (Fig. 2*B*), and used them in solid-phase assays. The assays showed that LOX binds to full-length FMOD and to the N-terminal fragment (FMODNT) but not to the truncated FMOD90 (Fig. 2, *C* and *D*). The binding to LOX is therefore sequestered to the N-terminal flank of FMOD, and the apparent *K_D_* of the FMOD-LOX interaction is ≅150 nm (Fig. 2*E*). We also analyzed whether tyrosine sulfation on FMOD could affect this interaction. Here we used bacterially expressed FMOD (lacking tyrosine sulfation) to assess its affinity for LOX. As Fig. 2*F* shows, the apparent affinity of LOX for FMOD with or without tyrosine sulfation is similar. Lastly, we also compared the relative affinities of FMOD and LOX for collagen. In our assay, LOX affinity for collagen was low, even at high molarity, whereas FMOD affinity for collagen had an apparent *K_D_* of ≅ 250 nm (Fig. 2*G*). We also tested whether FMOD could increase or inhibit LOX-collagen affinity, but the data were inconclusive (data not shown).

##### Lysyl Oxidase in Fibromodulin-null Mice

**FIGURE 3.**
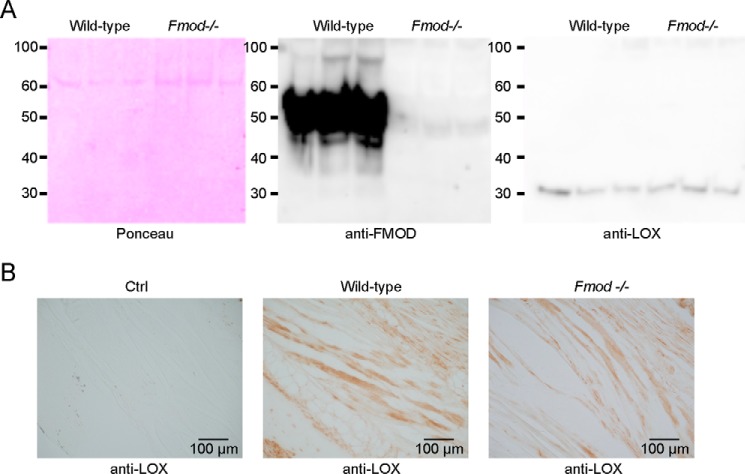
**Lysyl oxidase quantity and distribution in *Fmod*^−/−^ mice.** *A*, guanidine-extracted proteins from wild-type and *Fmod*^−/−^ tail tendons were precipitated with ethanol, run on SDS-PAGE, transferred to a nitrocellulose membrane, and stained with Ponceau or detected with anti-FMOD or anti-LOX antibodies (*n* = 3 biological replicates). *B*, immunodetection of LOX was performed on formalin-fixed tail tendons of wild-type and *Fmod*^−/−^ mice. The sections were deparaffinized, hydrated, and processed for antigen retrieval and peroxidase quenching before blocking with 10% goat serum and incubating with rabbit anti-LOX (or isotype control (*Ctrl*) antibody) followed by HRP-conjugated anti-rabbit. The staining was developed using diaminobenzamidine peroxidase.

Because we detected an interaction between FMOD and LOX, we then investigated whether LOX processing, distribution, or quantity was altered in *Fmod*^−/−^ mice. We used guanidine extracts from tail tendon tissue from wild-type and *Fmod*^−/−^ mice to immunoblot for LOX. We could not detect any major deviation in quantity or processing of LOX in *Fmod*^−/−^ mice. The latter was evident from observing only a 32-kDa band (processed LOX) but no 55-kDa pro-LOX band in either mice (Fig. 3*A*). Staining for LOX in tail tendon sections of wild-type and *Fmod*^−/−^ mice did not reveal any obvious changes in the distribution of LOX (Fig. 3*B*).

##### Lysyl Oxidase Activity Influenced by the N-terminal Flank of Fibromodulin

**FIGURE 4.**
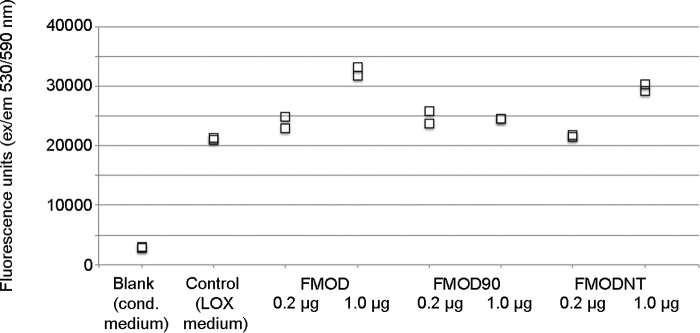
**Lysyl oxidase activity in the presence of fibromodulin.** Lysyl oxidase activity was measured with a commercial kit. As a sample, conditioned (*cond*) medium from 293T cells expressing LOX (or from LOX-negative control cells) was used. The medium was preincubated for 1 h with FMOD or its variants and then mixed with the substrate and incubated for 15 min at 37 °C. Enzyme activity was measured by fluorescence emission at 590 nm after excitation at 530 nm. The plotted values are biological duplicates. Similar results were obtained in three independent experiments.

Next we wanted to investigate whether the presence of FMOD could stimulate or inhibit LOX activity. Unfortunately, we could not measure LOX activity directly in protein extracts from mouse tendons because of a very low signal. We therefore used conditioned cell culture medium from 293T cells overexpressing LOX and mixed it with different amounts of FMOD, FMOD90, or FMODNT. Here we used fresh total cell culture medium from cells expressing LOX because we observed that purification of this enzyme dramatically decreased its activity, as reported previously (38). Fig. 4 shows that addition of 1 μg of full-length FMOD or the N-terminal part of FMOD to LOX-containing medium can increase LOX activity. FMOD gives a 62% (*p* < 0.05) and FMODNT gives a 47% (*p* < 0.05) increase, whereas the truncated FMOD90 protein can achieve this effect only marginally (18%, not significant).

## Discussion

We reasoned that FMOD could function as a LOX modulator during collagen fibrillogenesis because *Fmod*^−/−^ mice tendons have an altered cross-linking pattern (11) and FMOD modulates collagen fibrillogenesis, *i.e.* when LOX is also recruited to collagen (1, 6, 25). Here we present data that show an FMOD interaction with collagen cross-linking sites as well as an interaction of FMOD with LOX that results in increased LOX activity. This, together with the mentioned reports, supports a previously unknown role of FMOD as a LOX-modulating protein that can influence site-specific cross-linking of collagen.

FMOD, along with other homologous small leucine-rich proteins, has tissue-specific modulatory functions during collagen fibrillogenesis, these functions being evident in the different SLRP-deficient mice (8, 39). Modulating fibril assembly could in itself secondarily influence the LOX-mediated collagen cross-linking, but our data show that FMOD can exert this effect through direct binding to some of the collagen helix cross-linking sites, of which there are two on each of the fibrillar collagens, represented here by peptides II-5, II-52, and III-5/6 and III-52/53 (Fig. 1, *A*, *B*, and *D*). Although all carry the putative KGHR binding sequence, the Toolkit peptides are not equivalent in the binding assays. III-6 and III-52 have low thermal stability (33, 34), 30 °C and 39 °C, respectively, compared with 47 °C for the Toolkits as a whole,^6^ perhaps accounting for their low FMOD binding. We have observed that the N-terminal site (peptide III-5) appears to have a higher affinity for FMOD, as judged from the higher inhibitory effect of peptide 5 on the FMOD-collagen interaction (Fig. 1*C*). This might be explained by the influence of the neighboring amino acids and suggests a fine-tuned mechanism of cross-link modulation. We also observed that the lysine in the KGHR sequence is essential for binding of FMOD to the cross-linking site (Fig. 1*F*), suggesting also that FMOD may not bind to an already cross-linked site, *i.e.* when lysine has reacted with a telopeptide allysine. This would suggest FMOD involvement in collagen fibril assembly before cross-linking takes place.

**FIGURE 5.**
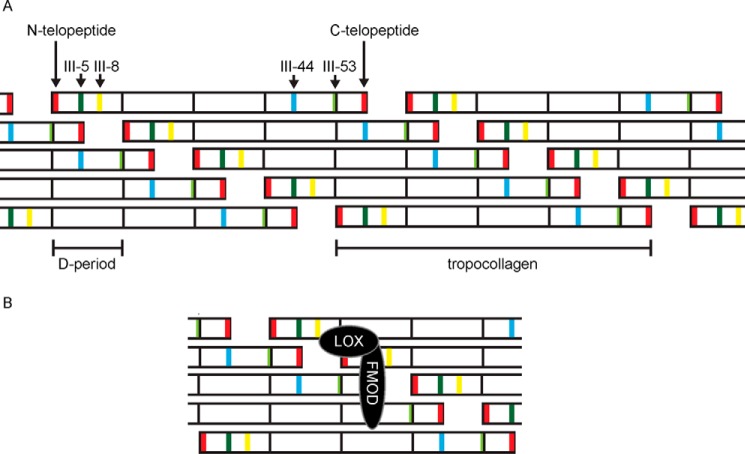
**Proposed model for collagen cross-linking modulation by fibromodulin.** *A*, alignment of the collagen III monomers is derived from 234-residue D periods. The identified high-affinity FMOD-binding sites in collagen are marked in *dark green* (III-5), *yellow* (III-8), *blue* (III-44), and *light green* (III-53). D periods are delimited with *black lines*. Collagen monomers are arranged in quarter-stagger, which reveals vertical alignment of III-44 and III-5 sites on neighboring collagen monomers. The two telopeptides (*red*) also align with FMOD-binding sites. *B*, proposed binding of the FMOD-LOX complex to a microfibril. FMOD binds across three monomers, interacting with the vertically aligned collagen-binding sites. LOX binds to the N-terminal end of FMOD and is positioned against the N-telopeptide in the microfibril.

We also observed that FMOD binds peptides previously identified as MMP-binding (peptides II-44 and III-44; Fig. 1, *A* and *B*) (35, 36) a site in both collagens that is adjacent to the cross-linking site on a neighboring tropocollagen molecule in a quarter-stagger assembled microfibril (Fig. 5*A*). Similarly, both these sites align with C-telopeptides (Fig. 5) that are excessively oxidized by LOX in *Fmod*^−/−^ mice (11). The span across the three sites (three tropocollagens) is ∼5 nm, which is roughly the size of the concave face of the LRR pocket in SLRPs (40, 41), and it is tempting to suggest a multivalent binding of FMOD to two interaction sites (III-5 and III-44) across the three collagen helices (as proposed in Fig. 5*B*). Therefore, FMOD could modulate collagen cross-linking by binding or possibly cross-bridging three collagen monomers around their cross-linking sites. Against this conclusion is the lack of potentiation of the inhibitory effect of both III-5 and III-44 on the FMOD-collagen interaction. On the other hand, inhibition by III-5 alone is very efficient, and any additive effect of III-44 may be marginal and hard to detect. Alternatively, bound FMOD could switch to an adjacent collagen monomer at some critical point during fibrillogenesis, thus switching from regulation of, perhaps, lateral assembly of collagens to regulation of cross-linking. The discrepancy between solid-phase binding and the ability of peptides to inhibit FMOD binding to collagen is intriguing. Peptide III-5 supports strong FMOD binding and, in solution, blocks FMOD binding to collagen I. In contrast, although II-44 and III-44 are also strong binders of FMOD, both only slightly inhibit its binding to collagen I. This suggests two binding modes, perhaps separate collagen-binding sites on FMOD; a first, high-affinity site that recognizes the KGHR sequence and a second remote site that recognizes the MMP interaction motifs in II-44 and III-44. Whether allosteric or co-operative effects allow these sites to regulate one another remains to be established.

Several other proteins besides FMOD, including fibronectin, fibrin, MMPs, and collagen C-telopeptide, bind to II/III-44 or its homologous site on collagen I (42–45). A cell might use such an overlapping interaction hot spot to differentially regulate collagen fibrillogenesis, cell adhesion, or contraction. Within the sequence of II-44 (Fig. 1*E*), binding of MMP13 to the canonical ¾-¼ cleavage site required residues spanning the first leucine and second arginine (35, 36), and although the only residues critical for MMP1 binding were the two leucines, contacts were observed in the MMP1 peptide co-crystal, including the next hydroxyproline (35, 36), whereas fibronectin binding embraces the first and second arginine residues (44). In summary, the sites in collagens I and II that bind MMPs, fibronectin, and FMOD are not identical but all overlap, and it would be anticipated that their binding would be mutually exclusive.

To speculate on one example, C-telopeptide binding to the site that corresponds to II-44/III-44 stimulates collagen fibrillogenesis (42, 46). FMOD could, through binding to this site, delay fibrillogenesis that is stimulated by the C-telopeptide. In this manner, the C-telopeptide oxidation by LOX would also be delayed because LOX activity increases on assembled collagen fibrils (25). Such a mechanism could explain the increased C-telopeptide oxidation in *Fmod*^−/−^ mice (11), *i.e.* the absence of FMOD would allow for a faster, uncontrolled, C-telopeptide-driven fibrillogenesis, thus triggering LOX to excessively oxidize C-telopeptides.

Binding of FMOD to II-44/III-44 also supports the previous observation that FMOD can inhibit MMP cleavage of collagen (47). Such an interaction could protect collagen from being digested before it starts to assemble into fibrils. FMOD could of course also protect the already assembled fibers by shielding the II-44 site from MMP.

Our interaction data are largely in line with the previously reported interactions of FMOD with collagen I CNBr fragments (48). In this report, FMOD bound collagen fragment CB6 (which includes a sequence from Toolkit peptide III-53), CB7 (includes III-44), and CB8 (includes III-8) but did not bind CB5, which contains the N-terminal helical cross-linking site. The explanation for this discrepancy could be a conformational unfolding of the KGHR sequence. KGHR is preceded by a methionine where CNBr cleaves, and the cleavage would leave the KGHR site without flanking, helix-inducing G*X*Y residues, likely to cause fraying of the new N-terminal end of the peptide, disrupting the binding site.

In the same paper (48), the authors also report on FMOD binding to collagen II CNBr fragments. The CB9,7 fragment containing the II-52 sequence was not used in the binding assay. One apparent discrepancy with our data is the absence of binding to CB10 (which contains the II-44 sequence) and binding to CB11 (where did not find any binding). Here one should consider that collagen II is more glycosylated, whereas Toolkit peptides are not; this could affect the FMOD-collagen II interaction. Our data, as discussed above, point to conserved interaction sites across collagens I, II, and III (Fig. 1*E*).

The binding of FMOD to collagen cross-linking sites suggested that FMOD might interact with the cross-linking enzyme LOX. Indeed, we were able to identify their interaction in a fibroblast cell culture using proximity ligation assays (Fig. 2*A*). We mapped this interaction to the N-terminal 12 amino acids of FMOD (Fig. 2, *B–E*) and observed no apparent effect of FMOD tyrosine sulfation on the interaction (Fig. 2*F*). We also observed that the binding of LOX to FMODNT was less pronounced than binding to FMOD (Fig. 2*E*). We think this could be due to a minor misfolding of FMODNT.

Our data suggest that FMOD forms a complex with LOX and collagens, where LOX binds at one end of FMOD (as shown here) and collagen(s) bind in the LRR domain (37). We did, however, observe that FMOD increased LOX activity even in the absence of collagen and that this activity depended on the N-terminal tail of FMOD (Fig. 4). This activity increase may be considered marginal, and possibly we could have had obtained a more potentiating effect had we used purified lysyl oxidase rather than cell medium. In our hands though, purification or storage of LOX was detrimental to its activity (although its interaction with FMOD remained unaffected), and therefore we used fresh cell medium for the activity assay. There may, of course, be other components in the cell medium that affect the extent of FMOD-stimulated LOX activity.

We suggest a model of FMOD-regulated collagen cross-linking where FMOD restricts premature fibril assembly and dysregulated cross-linking of collagens. This is achieved by binding to the surface of a collagen microfibril at its cross-linking site while simultaneously binding LOX and directing its activity toward a specific lysine residue on an adjacent microfibril (Fig. 5*B*). Such an interaction may explain the tissue-specific cross-linking found in collagen matrices populated by FMOD, including tendons, ligaments, or fibrotic tissues. One should keep in mind that LOX-FMOD interaction does not ensure LOX-mediated cross-linking but appears to direct it toward specific collagen lysine residues.

Previously we reported excessive oxidation of collagen C-telopeptides in *Fmod*^−/−^ mice (11). We did not detect any changes in LOX processing or quantity in *Fmod*^−/−^ tendons, but we show here that FMOD interacts with LOX, changes its activity, and binds to collagen cross-linking sites. We reason that FMOD, by binding across the C-telopeptides, could shield LOX from excessively oxidizing telopeptides within one microfibril, a reaction that would prompt uncontrolled cross-linking between C-telopeptide allysines, forming aldols interlinking other microfibrils. In FMOD-controlled packing and cross-linking of microfibrils, LOX could be directed toward the telopeptides of another microfibril by binding to one end of FMOD, triggering the formation of a specific interfibril cross-link. It is also possible that LOX becomes positioned toward N-telopeptides in the same microfibril (Fig. 5*B*). This would ensure concerted, tissue-specific packing of microfibrils into fibrils and regulated intrafibrillar cross-linking.

In summary, our current and previous data show that FMOD can modulate LOX activity on specific sites on collagen and support a novel theory regarding control of specific collagen cross-linking through collagen-associated proteins, likely crucial in the proper assembly of tendons but also of fibrotic collagen matrices. The exact cross-links being formed in healthy and fibrotic tissues remain to be shown, as does the identity of the collagen-associated proteins that modulate their formation. This effort could open new perspectives and reveal new targets for treating fibrotic conditions.

## Author Contributions

S. K. conceived the study, designed, performed, and analyzed the experiments, and wrote the paper. R. W. F. designed the Toolkit peptides, synthesized by D. B. and A. B., and revised the manuscript with S. K.. K. R. analyzed the experiments and revised the manuscript with S. K. All authors reviewed the results and approved the final version of the manuscript.

## Footnotes

6 D. A. Slatter, unpublished data.

## Footnotes

5 The abbreviations used are:

FMOD: fibromodulin
LOX: lysyl oxidase
Ni-NTA: nickel-nitrilotriacetic acid
MMP: matrix metalloproteinase
SLRP: small leucine-rich proteoglycan
CNBr: cyanogen bromide.
